# Syntaxin 6: A novel predictive and prognostic biomarker in papillary renal cell carcinoma

**DOI:** 10.1038/s41598-019-39305-z

**Published:** 2019-02-28

**Authors:** Taylor C. Peak, Yixin Su, Andrew G. Chapple, Jacqueline Chyr, Gagan Deep

**Affiliations:** 1 Department of Cancer Biology, Wake Forest School of Medicine, Winston-Salem, North Carolina USA; 2 Biostatistics Program, School of Public Health, LSU Health Sciences Center, New Orleans, LA USA; 3 School of Bioinformatics, University of Texas Health Science Center, Houston, Texas USA; 4 Department of Urology, Wake Forest School of Medicine, Winston-Salem, North Carolina USA; 5 Wake Forest Baptist Comprehensive Cancer Center, Wake Forest School of Medicine, Winston-Salem, North Carolina USA

## Abstract

Syntaxin 6 is a SNARE family protein known to play an important role in intracellular trafficking. Here, we examined the tumorogenic role of syntaxin 6 in renal cell carcinoma (RCC). The Cancer Genome Atlas (TCGA) was queried for clinicopathologic data and syntaxin 6 expression. We found a significant difference in overall survival (OS) between groups, with high syntaxin 6 expression correlating with decreased survival. When stratifying the data based on histological subtype, the papillary RCC subtype exhibited a significant correlation between syntaxin 6 expression and survival. Using ROC curve, we calculated the area under the curve (AUC) to determine the ability of syntaxin 6 to predict 3-year overall survival. The AUC for syntaxin 6 was 0.73, significantly higher compared to 0.52 for T stage. Next, syntaxin 6 expression was evaluated in clear cell (786-O and Caki-1) and papillary (Caki-2 and ACHN) RCC cells. Syntaxin 6 expression was higher in Caki-1 and ACHN RCC cells. Silencing of syntaxin 6 in ACHN cells significantly decreased the cell viability (p < 0.001). Overall, syntaxin 6 could be a prognostic biomarker for patients with papillary RCC and syntaxin 6 inhibitors hold promise as a novel therapy against RCC.

## Introduction

Renal cell carcinoma (RCC) has the highest mortality of all urologic malignancies. In 2017, 63,990 individuals developed RCC, and about 14,400 died due to this malignancy^1^. It is a heterogeneous disease that can be divided into multiple histological subtypes, encompassing clear cell (ccRCC), papillary (pRCC), chromophobe (chRCC), collecting duct, and unclassified subtypes^2^. Of the three major histologic subtypes of RCC, 70–80% of tumors are ccRCC, 10–20% are pRCC, and 5–10% are chRCC.

Because the majority of cases of RCC present as the ccRCC subtype, researchers have gained a relatively better understanding of its pathogenesis. Classically, mutations in the von Hippel-Lindau (VHL) gene prevents ubiquitination and degradation of hypoxia-inducible factors (HIF) such as HIF-1α^3^. This in turn leads to increased expression of vascular endothelial growth factor (VEGF), and resulting angiogenesis, as well as tumor formation and progression. pRCC and chRCC subtypes have been studied to a lesser extent, and what we do know is based largely on familial forms of the disease^3,4^. In light of this, many of the therapies developed to treat advanced RCC have been based on disrupting the known pathways of ccRCC^5^. Current therapies include mTOR inhibitors and tyrosine kinase inhibitors, both central to the HIF1α and VEGF pathway^6^.

Despite advances in developing these targeted therapies, overall survival in advanced, metastatic RCC cases remains dismal, often less than 20 months^7^. In order to create more effective therapies, we must explore novel cellular pathways that may be involved in the progression of disease. Intracellular vesicle trafficking and specifically the soluble N-ethylmaleimide attachment protein receptor (SNARE) proteins, have been shown to be integral to tumor development, progression, and metastasis^8^. SNARE proteins are tail-anchored membrane proteins involved in membrane fusion events along the secretory pathway^8^. There are two types of SNAREs, classified as t-SNAREs and v-SNAREs, with t-SNAREs being found on the target membrane and v-SNAREs on the vesicle membrane. Of the t-SNARE proteins, syntaxin 6 is among those particularly important in vesicle fusion. Syntaxin 6 is primarily located on the trans-Golgi network, with the remaining protein located in endosomes and transport vesicles^9^. Under conditions of overexpression, it can be found diffusely spread within the cytoplasm^10^. Gene expression analyses have demonstrated that syntaxin 6 is upregulated in numerous cancers including breast, colon, liver, pancreatic, prostate, bladder, skin, testicular, tongue, cervical, lung and gastric cancers^11^. In cancerous cells, syntaxin 6 has been shown to regulate chemotactic cell migration through integrin trafficking to the cell surface^11^. Syntaxin 6 has also been identified as a common transcriptional target of p53 family members (p53, p63 and p73)^12^. In the present study, we set out to determine syntaxin 6 role in predicting oncologic outcomes in those patients with RCC, as well as its therapeutic value as a targetable protein.

## Results

### Syntaxin 6 expression in various sub-types of RCC

**Figure 1:**
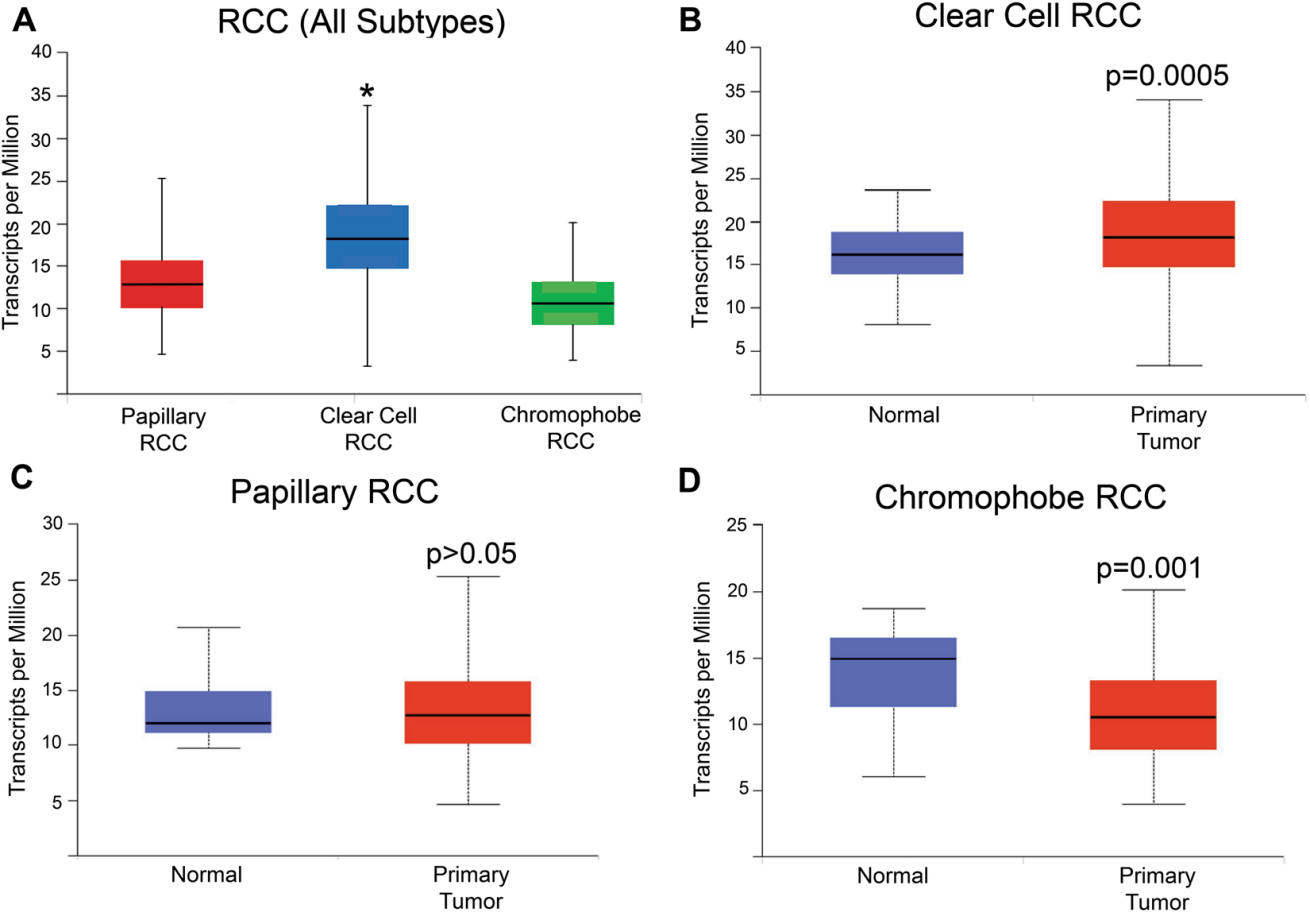
Syntaxin 6 mRNA expression in RCC subtypes. (**A**) Comparison of syntaxin 6 mRNA expression in all 3 histologic subtypes of renal cell carcinoma. (**B–D**) Comparison of syntaxin 6 mRNA expression in normal tissue and primary tumors (ccRCC, pRCC and chRCC). Graphs were generated using the UALCAN interactive software to analyze the TCGA database. *p < 0.05.

We first queried the TCGA-Renal Cell Carcinoma database for syntaxin 6 mRNA expression. Because of the differences in disease progression based on the histologic subtypes of RCC, we decided to stratify the dataset based on the three most common subtypes, ccRCC, pRCC, and chRCC. The median expression of syntaxin 6 for each subtype, as well as for normal tissue controls, was calculated (Fig. 1A–D). Patients with ccRCC had significantly higher median expression of syntaxin 6 (18.163) than patients with pRCC (12.74) or chRCC (10.518) sub-groups (p < 0.0001). Furthermore, ccRCC tumors had significantly higher syntaxin 6 expression as compared to normal kidney (p = 0.0005). This was not true with pRCC (p > 0.05). chRCC had significantly less syntaxin 6 expression as compared to normal kidney (p = 0.001).

### Syntaxin 6 expression predicts survival in RCC patients

**Figure 2:**
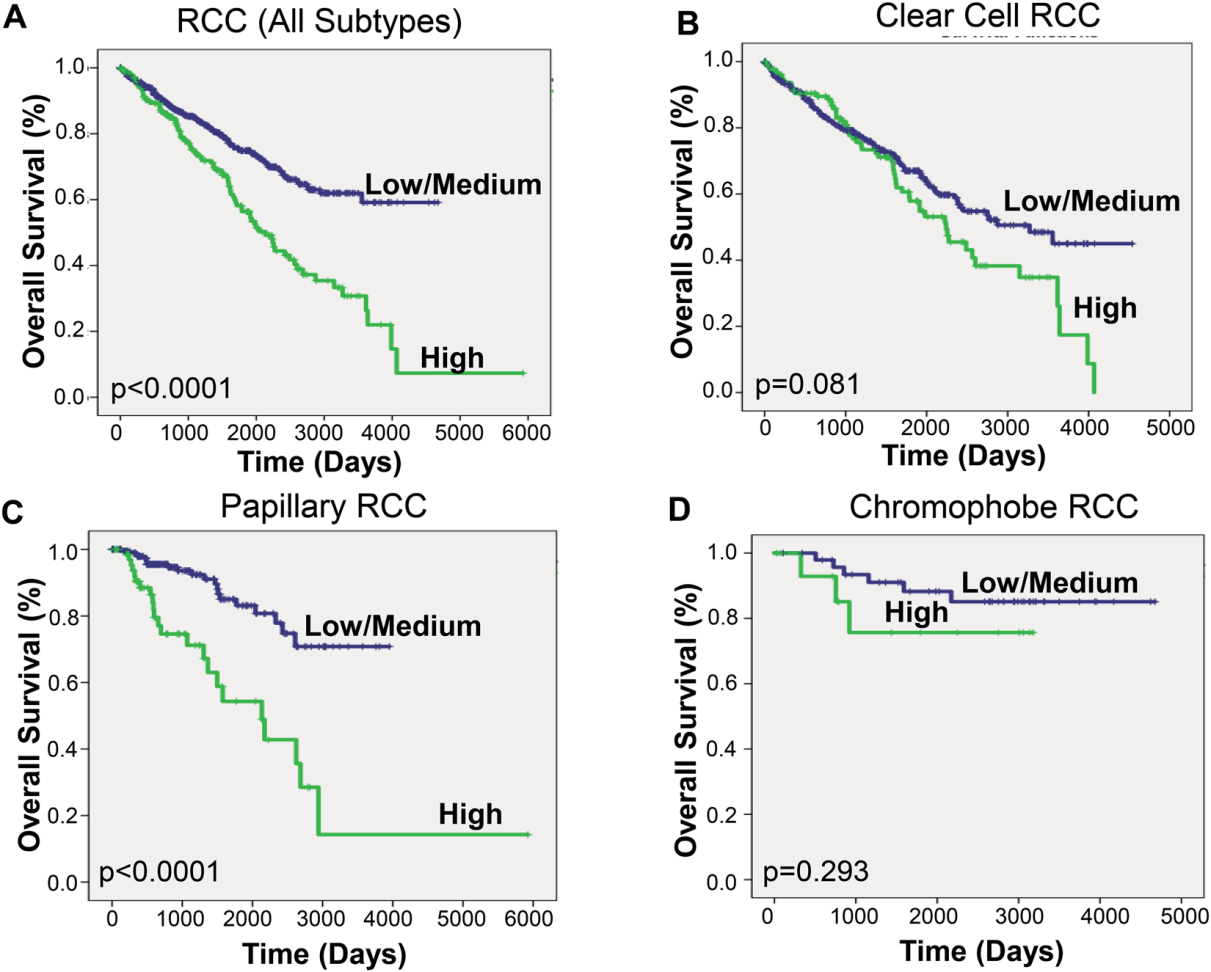
Kaplan-Meier analysis of RCC histologic subtypes based on syntaxin 6 expression. The entire RCC patient cohort was divided into quartiles based on syntaxin 6 expression. The cohort was then stratified into 2 groups: High expression (top quartile) Low/Medium (<top quartile). Kaplan-Meire curve was then generated based on overall survival for all renal cell carcinoma patients (**A**) and then stratified for each histologic subtype- pRCC, ccRCC and chRCC (**B–D**).

We then determined cut-points for low/medium vs high syntaxin 6 expression in the entire RCC cohort as a whole, and then separately based upon histologic subtype. The high expression group represented values in the top quartile; the low/medium group represented values below the top quartile. Next, we performed Kaplan-Meier analysis for overall survival using the entire RCC database, stratifying the results based on the low/medium and high syntaxin 6 expression sub-groups (Fig. 2A). We found that there was a significant difference in overall survival between the two groups (p < 0.0001). Median overall survival for the entire cohort was 9.740 years. Those patients with high syntaxin 6 expression had a median overall survival of 5.844 years. Because the survival curve for low/medium expression did not drop below 50%, the median survival time for this group could not be calculated.

Interestingly, after analyzing survival based upon histologic subgroup, the correlation between syntaxin 6 expression and overall survival remained decreased in those patients with only pRCC. Median overall survival for the entire ccRCC cohort was 7.126 years. The median follow-up time was 3.288 years, with a range between 0 and 12.430 years. Median overall survival for ccRCC tumors with high syntaxin 6 expression was 6.140 years. This was non-significantly lower than survival for those patients with low/medium syntaxin 6 expression, which was 8.962 years (p = 0.081) (Fig. 2B). Similarly, median overall survival for pRCC tumors with high syntaxin 6 expression (5.844 years) was significantly lower than survival for those tumors with low/medium syntaxin 6 expression (p < 0.0001) (Fig. 2C). Median follow-up time for the pRCC cohort was 2.1 years, with a range between 0 and 16.233 years. Once again the survival curve for low/medium expression did not drop below 50%, and thus the median survival time for this group could not be calculated. However, there was no significant trend observed in chRCC (Fig. 2D).

**Figure 3:**
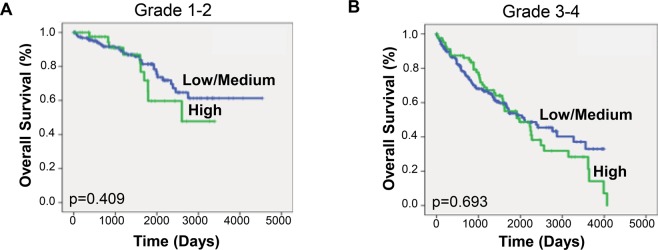
Kaplan-Meier analysis of clear cell RCC according to grade. Kaplan-Meier curve was generated based on overall survival for ccRCC patients with low/medium and high syntaxin 6 expression; it was then stratified based on low Fuhrman grade (1–2) and high Fuhrman grade (3–4) histology.

**Figure 4:**
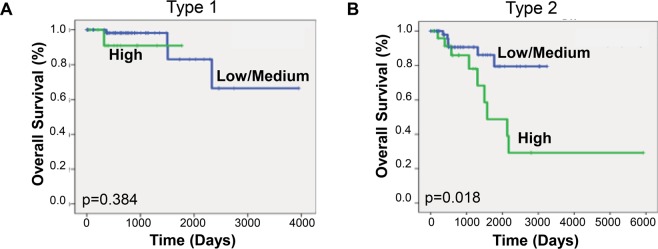
Kaplan-Meier analysis of papillary RCC according to type 1 vs type 2. Kaplan-Meier curve was generated based on overall survival for pRCC patients with low/medium and high syntaxin 6 expression; it was then stratified based on type1 and type 2 tumors.

Studies have shown that other histologic features within both ccRCC and pRCC cohorts are predictors of disease aggressiveness and overall survival^13^. Therefore, we separated the ccRCC cohort into low grade (Furhman 1–2) and high grade (3–4) tumors and performed Kaplan-Meier analysis. We did not find a significant difference in overall survival in either group when comparing syntaxin 6 expression (Fig. 3). A similar analysis was performed on the pRCC cohort, instead looking at whether the tumors were type 1 or type 2. Type 2 tumors are recognized as the more aggressive pRCC phenotype associated with most deaths^14^. While syntaxin 6 expression was not significantly associated with survival in patients with Type 1 pRCC tumors, it was significantly associated with a decrease in overall survival for Type 2 patients (Fig. 4).

### Correlation between syntaxin 6 and clinical-pathological features

The ccRCC and pRCC cohorts were classified into different groups according to the clinical and pathological features, with the objective to discover the association between syntaxin 6 gene expression and the clinical and pathological features. In the ccRCC cohort, higher syntaxin 6 expression was closely associated with male gender (p < 0.0001), higher grade (p = 0.002), and higher pathologic T stage (p = 0.031); however, it was not associated with age or race (white vs non-white) (Table 1). We found that across tumor grade (1–4) (p < 0.0001), higher syntaxin 6 expression was correlated with worse overall survival (data not shown). This did not hold true for gender (p = 0.28) or race (*P* = 0.83) (data not shown). In the pRCC cohort, higher syntaxin 6 expression was closely associated with the female gender (p*˂*0.008) and higher pathologic T stage (p < 0.0001); however, it was not associated with age, tumor type (Type 1 vs Type 2), or race (white vs non-white) (Table 2). We found that across race (p < 0.0001), gender (p < 0.0001), and body weight (p < 0.0001), higher syntaxin 6 expression was correlated with worse overall survival (data not shown).

**Table 1:** Association of syntaxin 6 expression with clinicopathological characteristics in clear cell RCC.

	Variable	Low/Medium Syntaxin 6	High Syntaxin 6	Total	p-Value
Age	≤65 >65	246 (61.5%) 154 (38.5%)	87 (65.4%) 46 (34.6%)	333 200	0.470
Gender	Male Female	239 (59.8%) 161 (40.2%)	106 (80.0%) 27 (20.0%)	345 188	<0.0001
Pathologic T Stage	1-2 3-4	267 (66.8%) 133 (33.2%)	75 (56.4%) 58 (43.6%)	342 191	0.031
Grade	1-2 3-4	197 (50.1%) 196 (49.9%)	46 (34.8%) 86 (65.2%)	243 282	0.002
Race	White Non-White	343 (93.2%) 25 (6.8%)	119 (95.2%) 6 (4.8%)	462 31	0.428

**Table 2:** Association of syntaxin 6 expression with clinicopathological characteristics in papillary RCC.

	Variable	Low/Medium Syntaxin 6	High Syntaxin 6	Total	p-Value
Age	≤65 >65	128 (58.7%) 90 (41.3%)	48 (66.7%) 24 (33.3%)	176 114	0.266
Gender	Male Female	170 (78.0%) 48 (22.0%)	44 (61.1%) 28 (38.9%)	214 76	<0.008
Pathologic T Stage	1-2 3-4	187 (86.2%) 30 (13.8%)	39 (54.9%) 32 (45.1%)	226 62	<0.0001
Tumor Type	1 2	64 (52.0%) 59 (48.0%)	13 (33.3%) 26 (66.6%)	77 85	0.124
Race	White Non-White	156 (83.4%) 31 (16.6%)	50 (89.3%) 6 (10.7%)	206 37	0.396

We found on univariate Cox proportional hazards regression that syntaxin 6 expression was not significantly associated with worse overall survival with hazard ratio of 1.326 (95%CI 0.965–1.822; p = 0.082) for ccRCC (Table 3). However, it was found to be significant in the pRCC cohort with hazard ratio of 4.057 (95%CI 2.243–7.341; *p* < 0.0001). We performed a multivariate analysis for ccRCC using pathologic stage, tumor grade, and age, and found that older age (HR 1.782; 95%CI 1.314–2.418; *p* < 0.0001), higher grade (HR 1.505; 95%CI 1.037–2.184; p = 0.032), and pathologic stage III (HR 2.060; 95%CI 1.346–3.153; p = 0.001) and stage IV (HR 4.848; 95% CI 3.197–7.351; p < 0.0001) disease remained significant predictors of overall survival (Table 3). For the pRCC cohort, we performed a multivariate analysis using pathologic stage and syntaxin 6 expression (Table 4). We found on analysis of the pRCC cohort that syntaxin 6 high expression (HR 2.779; 95%CI 1.392–5.549; p = 0.004), along with pathologic stage III (HR 2.783; 95%CI 1.251–6.192; p = 0.012) and stage IV (HR 7.511; 95%CI 2.996–18.832; p < 0.0001) disease, remained significant predictors of overall survival (Table 4).

**Table 3:** Univariate and Multivariate Cox proportional hazards model predictors of overall survival in clear cell RCC.

Variable		Univariate			Multivariate		
		HR	95% CI	p-Value	HR	95% CI	p-Value
Syntaxin 6	Low (Reference) High	1.326	0.965-1.822	0.082	—	—	—
Age	≤65 (Reference) >65	1.590	1.181-2.140	0.002	1.782	1.314-2.418	<0.0001
Gender	Male (Reference) Female	0.988	0.725-1.346	0.938	—	—	—
Race	White (Reference) Other	1.128	0.554-2.298	0.740	—	—	—
Grade	1-2 (Reference) 3,4	2.521	1.795-3.542	<0.0001	1.505	1.037-2.184	0.032
Pathologic Stage	I (Reference) II III IV	1.300 2.517 5.416	0.701-2.413 1.679-3.775 3.708-7.909	0.405 <0.0001 <0.0001	1.355 2.060 4.848	0.727-2.524 1.346-3.153 3.197-7.351	0.339 0.001 <0.0001

**Table 4:** Univariate and multivariate Cox proportional hazards model predictors of overall survival in papillary RCC.

Variable		Univariate			Multivariate		
		HR	95% CI	P-Value	HR	95% CI	P-Value
Syntaxin 6	Low (Reference) High	4.057	2.243-7.341	<0.0001	2.779	1.392-5.549	0.004
Age	≤65 (Reference) >65	0.900	0.497-1.632	0.730	—	—	—
Gender	Male (Reference) Female	1.407	0.733-2.701	0.305	—	—	—
Race	White (Reference) Other	0.908	0.402-2.055	0.818	—	—	—
Pathologic Stage	I (Reference) II III IV	0.879 1.932 4.486	0.196-3.945 1.932-8.656 4.486-25.864	0.866 <0.0001 <0.0001	0.963 2.783 7.511	0.214-4.326 1.251-6.192 2.996-18.832	0.963 0.012 <0.0001

### Syntaxin 6 as a biomarker to predict patient survival

**Figure 5:**
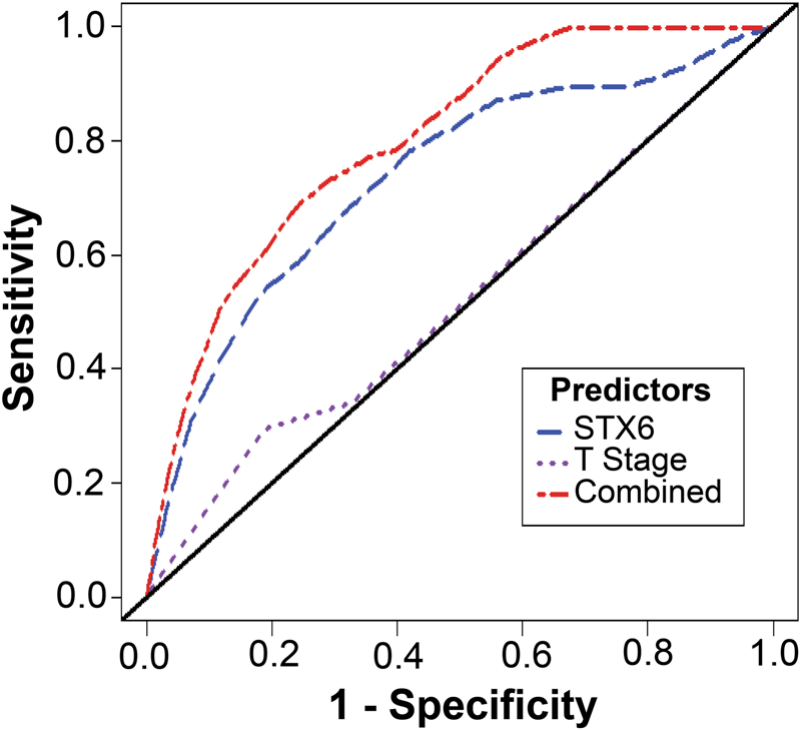
Multivariable prediction of 3-year overall survival in papillary RCC patients. A time-dependent receiver operating characteristic (ROC) analysis was performed to determine the sensitivity and specificity of 3-year overall survival prediction for syntaxin 6, pathologic T stage, and the combination of the two variables. The AUC score for the full dataset was calculated and then 1000 bootstrap samples of the AUC score were used to obtain a confidence interval for each AUC score.

We then performed receiver operating characteristic (ROC) analysis to determine the sensitivity and specificity of 3-year overall survival prediction for syntaxin 6 in patients with pRCC (Fig. 5). We also compared this to the pathologic T stage, and then combine the two variables (syntaxin 6 and T stage) to determine whether there was an increase in predictive value. For each biomarker, we found the AUC score for the full dataset and drew 1000 bootstrap sample datasets from the original data, computing the AUC score for each sample. This produced 95% bootstrap confidence intervals for the AUC score that we can use to determine if the covariates have significant differences in 3-year survival prediction ability. To analyze the combination of syntaxin 6 and T stage, we used the coxph function in R to obtain a model estimate predicting overall survival given these two covariates. The estimated coefficients in this model are used to obtain a single numerical value for each patient based on these two covariates and these transformed values are used to determine the AUC for both predictors.

As seen in Fig. 5, ROC curves indicated that the AUC of syntaxin 6 and T stage was 0.73 and 0.52, respectively. The 95% bootstrap confidence intervals for the AUC scores for syntaxin 6 and T stage did not overlap, indicating that syntaxin 6 is a significantly better (p < 0.05) predictor of 3-year survival than T stage. When using both syntaxin 6 and T stage as predictors, we found that the AUC increased to 0.80.

### Effect of syntaxin 6 co-expression with key RCC regulators on overall patient survival

**Figure 6:**
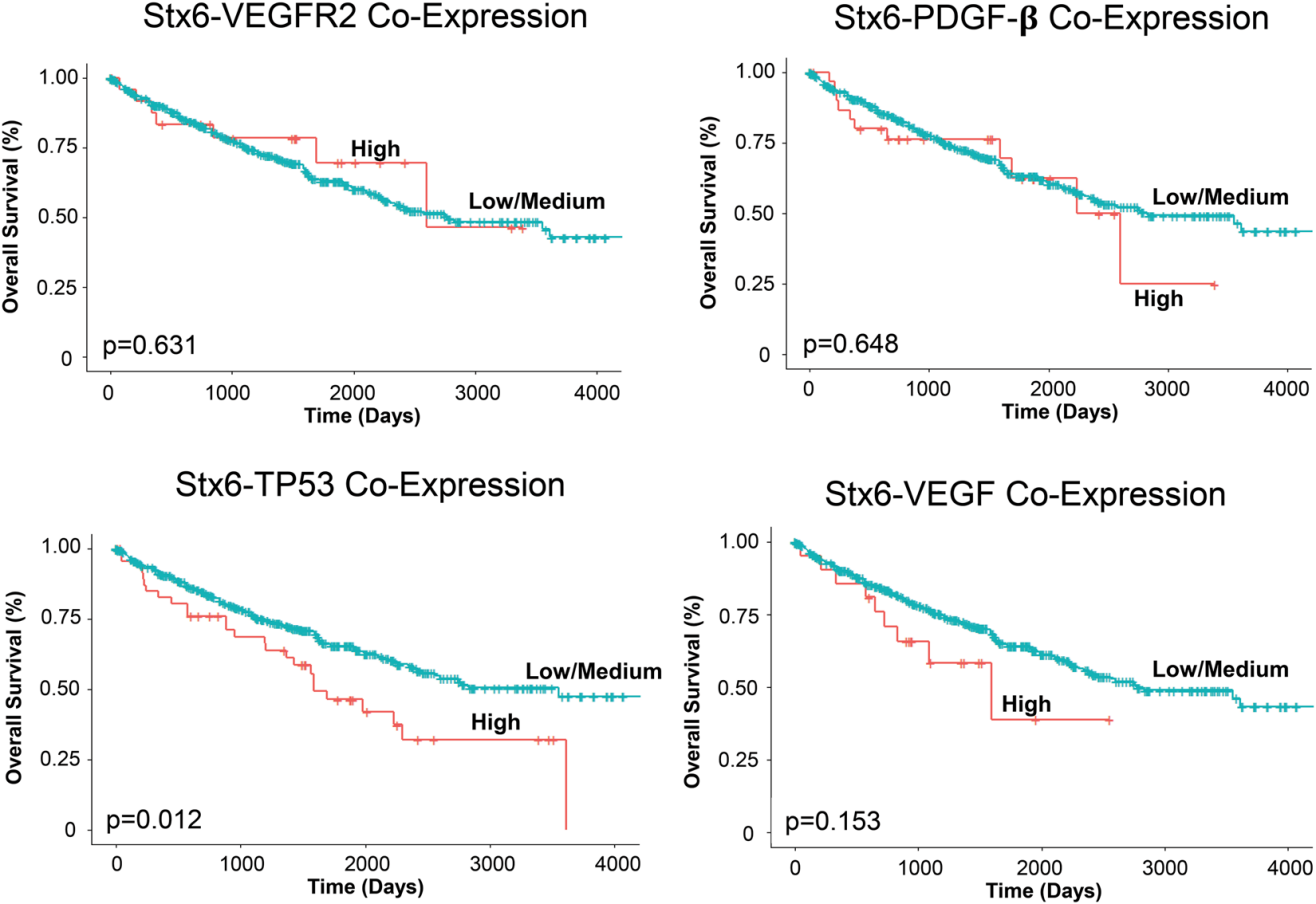
Kaplan-Meier analysis of clear cell RCC with evaluation of co-expression of syntaxin 6 and key genes. Syntaxin 6 co-expression with VEGFR2, PDGF-β, TP53, and VEGF in ccRCC patient cohort was stratified into 2 groups: High expression (top quartile) and Low/Medium (<top quartile) and overall survival was compared.

**Figure 7:**
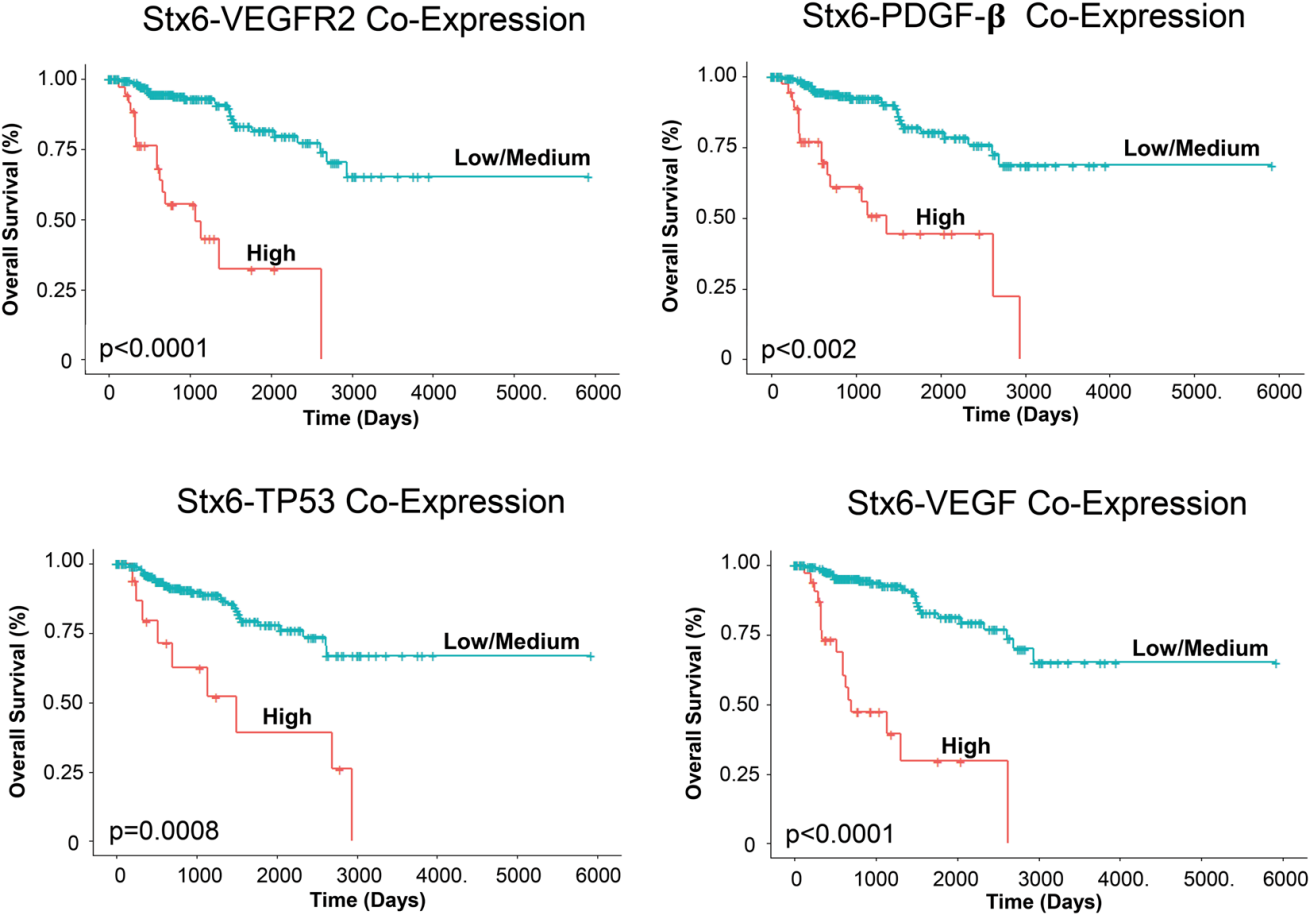
Kaplan-Meier analysis of papillary RCC with evaluation of co-expression of syntaxin 6 and pro-angiogenic genes. Syntaxin 6 co-expression with VEGFR2, PDGF-β, TP53, and VEGF in pRCC patient cohort was stratified into 2 groups: High expression (top quartile) and Low/Medium (<top quartile) and overall survival was compared.

We then evaluated the RCC patients survival outcomes based upon co-expression of several known RCC regulators and syntaxin 6. These regulators included VEGF, VEGFR2, TP53, and PDGF- β. In our analysis, we stratified those patients who had high expression of both syntaxin 6 and each of the above mentioned proteins (High group) and those who did not have high expression (Low/Medium group). High expression was considered to be above the first quartile. Our analysis only demonstrated a significant difference in survival of those patients with ccRCC who had high expression of syntaxin 6 and TP53 but not with VEGFR2, PDGF-β and VEGF (Fig. 6). Interestingly, high co-expression of syntaxin 6 with VEGFR2, PDGF-β, TP53 and VEGF all predicted significantly worse survival in those patients with pRCC (Fig. 7).

### Syntaxin 6 plays important role in cell survival in RCC

**Figure 8:**
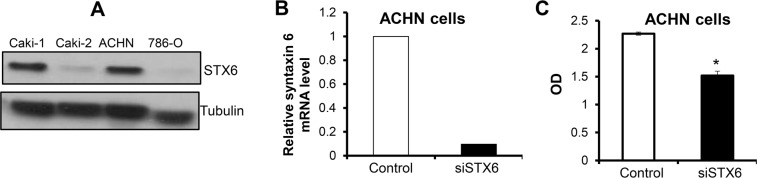
Syntaxin 6 is upregulated in metastatic RCC cell lines and regulates survival of metastatic papillary RCC cells. (**A**) Syntaxin 6 protein expression was measured by Western blotting in ccRCC (786-O and Caki-1) and pRCC (Caki-2 and ACHN) cell lines. Membrane was also probed for tubulin to assess protein loading. (**B**) ACHN cells were transfected with siRNA for syntaxin 6 as described in methods and syntaxin 6 knock-down was confirmed by real-time PCR. Relative syntaxin 6 mRNA level compared to GAPDH in control cells and syntaxin 6 knock-down is presented. **(C)** ACHN cells were transfected with siRNA for syntaxin 6 and cell viability was measured after 72 hrs in MTT assay. *p < 0.001.

Thus, after having demonstrated the predictive value of high syntaxin 6 expression in overall survival for those patients with pRCC, we next explored its expression in RCC cell lines as well as its potential role in cell survival. We utilized 4 RCC cell lines, of which included two ccRCC (786-O and Caki-1) and two pRCC cell lines (Caki-2 and ACHN). We found that all RCC cell lines expressed syntaxin 6 but with higher expression in Caki-1 and ACHN cells (Fig. 8A).

In order to demonstrate the importance of syntaxin 6 on cell survival, we knocked-down its expression in ACHN cells using specific syntaxin 6 siRNA. Syntaxin 6 knock-down was confirmed by real time-PCR (Fig. 8B). After confirming successful knock-down, we analyzed the effect of syntaxin 6 knockdown on cell viability. As shown in Fig. 8C, syntaxin 6 significantly reduced (by 33%, p ≤ 0.001) the viability of ACHN cells.

## Discussion

For those patients who present with large tumors and advanced disease, surgery alone is unlikely to provide a cure. These patients often require targeted therapy. Tyrosine kinase inhibitors (TKIs), mTOR inhibitors, and VEGF inhibitors have all been developed based on molecular understanding of the pathogenesis of ccRCC^6^. More recently researchers have begun developing therapeutic targets for pRCC and other non-ccRCC. With c-met mutations known to be common in pRCC, MET inhibitors such as capozantanib and foretinib have been developed and are in clinical trials^15,16^. Nevertheless, c-met mutations are specific to Type 1 pRCC, not the more aggressive Type 2 tumors. Still further, many of these agents, along with those approved for ccRCC, only provide a few months of additional survival advantage. Therefore, we must identify molecular markers that can be used to predict which therapies non-ccRCC will respond. Further by demonstrating the prognostic value of novel markers, we will be able to develop new agents that target molecule/s or oncogenic pathway/s different from currently known.

In this study, using TCGA database, we suggest that syntaxin 6 could serve as a potential biomarker for predicting overall survival mainly in pRCC patient. Of note, there are clinical limitations to the TCGA database. There is a limited number of clinicopathologic variables included within the database that could affect our multivariate analyses. Furthermore, RCC- specific treatment modalities were not provided. These treatments include targeted medical therapies, cytoreductive surgery, and metastatectomy, all of which are known to influence survival. Finally, disease-specific survival was not available, which would have otherwise provided a stronger biologic justification for syntaxin 6 use as a clinical biomarker.

The relationship between high syntaxin 6 expression and aggressive disease appears to be more strongly associated with type 2 pRCC tumors. This is particularly interesting given that type 2 pRCC tumors are associated with a greater stage, higher grade, higher frequency of necrosis and sarcomatoid features with worse outcome and more aggressive disease^14,17^. In addition, syntaxin 6 may also serve as a potential target for drug development, thus adding to the already expanding number of agents used to treat ccRCC, as well as to the very few agents that are being developed to treat non-ccRCC histology, such as pRCC. However, currently, specific inhibitors of syntaxin 6 are not available. Many neurotoxins such as botulinum and tetanus have been used as general inhibitors of various SNARE proteins. These inhibitors have shown efficacy against various cancer cell lines so these inhibitors could be tested for their efficacy against RCC cells^18^. Further, there is a need to screen chemical libraries for specific syntaxin 6 inhibitors.

The exact mechanism through which high syntaxin 6 is promoting survival in pRCC cell lines is unclear. However, it may be that it directly affects the regulation of VEGFR2 protein. Prior research has demonstrated the regulation of VEGFR2 by syntaxin 6^19^. They found that inhibition of syntaxin 6 function led to targeting of Golgi-localized VEGFR2 for degradation in lysosomes. VEGFR2 in turn was unable to migrate to the plasma membrane for promotion of subsequent angiogenesis, cell proliferation, cell migration, and vascular tube formation^19^. VEGFR2 is the major mediator of VEGFA- driven responses in endothelial cells and a pivotal signal transducer during both physiologic and pathologic angiogenesis. Moreover, VEGFR2 expression is significantly upregulated in the vascular endothelium of most common human solid tumors, including RCC^20^. Interestingly, despite the well-known HIF1α/VEGF pathway involved in ccRCC, one group found that the expression of VEGF, VEGFR2, and PDGF-β were all higher in pRCC compared to that of ccRCC^21^. These results are in agreement with those of an earlier study, which reported that VEGF protein expression was significantly higher in patients with pRCC than ccRCC, but did not differ between ccRCC and other RCC subtypes^22^. Although these proangiogenic growth factors are known to play critical roles in the development of vascular ccRCC through VHL mutations, it is surprising to note their relatively high expression in pRCC, where VHL mutations rarely occur. It is tempting to suggest the involvement of syntaxin 6 in promoting the higher expression of these angiogenesis-related molecular players in pRCC. Indeed, this aligns with our own co-expression TCGA data analysis; and higher co-expression of syntaxin 6 and pro-angiogenesis factors (VEGF, VEGFR2 or PDGF-β) in pRCC was associated with adverse prognosis.

Besides VEGFR2, syntaxin 6 role has been reported in trafficking of integrins and caveolin1^11^. Further, syntaxin 6 is an important regulator of plasma membrane structure and biophysical properties through delivery of several components of lipid rafts such as GM1 ganglioside (GM1), glycosylphosphatidylinositol (GPI) and caveolin-1^10,23^. Several studies have shown that lipid rafts could trap chemotherapeutic drugs^24,25^ and contribute in drug resistance. Further, lipid rafts are also known to regulate exosome biogenesis, and exosomes are enriched in lipid raft components and known to contribute in drug-resistance^26–29^. Syntaxin 6 role has been reported in exocytosis^30^. Therefore, syntaxin 6 could contribute in disease progression through intracellular trafficking of key oncogenes as well as through trapping or excretion of chemotherapeutic drugs and contributing in drug resistance. Thereby, higher syntaxin 6 could be associated with reduced overall survival in RCC patients.

It is important to understand the molecular reasons for higher syntaxin 6 expression in RCC. High syntaxin 6 expression has also been reported in numerous cancers including breast, colon, liver, pancreatic, prostate, bladder, skin, testicular, tongue, cervical, lung and gastric cancers^11^. One of the key regulators of syntaxin 6 expression is p53 family members (p53, p63 and p73)^12^. Importantly, p53 increased expression, but not the p53 mutation, is associated with reduced overall survival/more rapid disease progression in RCC^31^. Therefore, the higher p53 expression could contribute to higher syntaxin 6 expression in RCC. Interestingly, in both ccRCC and pRCC, higher co-expression of p53 and syntaxin 6 was associated with adverse prognosis.

In conclusion, present study suggests that syntaxin 6 is a novel biomarker to predict survival in RCC patients especially in pRCC patients. These results support clinical studies using pathologic specimens and prospectively-obtained clinical data to further validate syntaxin 6 use as a biomarker to assess RCC aggressiveness and patient’s survival. Furthermore, results from present study also suggest the need to better understand molecular mechanistic roles of syntaxin 6 in the survival of RCC cells as well as provide a rationale to develop and test syntaxin 6 inhibitors as a novel therapy against RCC.

## Materials and Methods

### TCGA Database

RNA sequencing (RNA-Seq) data from 973 individuals within the Pan-Kidney cohort (KICH + KIRC + KIRP) were obtained from the TCGA data portal (https://gdc.cancer.gov/). Overall, 889 Pan-Kidney patients were included in this study, including data from 533 KIRC, 290 KIPAN, and 66 KICH tumor tissue samples, along with 72 non-tumorous adjacent-normal kidney tissue samples up to August 17, 2017. Clinical and pathologic data on TCGA patients were also downloaded from the Broad Institute TCGA GDAC Firehose. In addition, the UALCAN (http://ualcan.path.uab.edu/index.html) was used as an interactive web resource to aid in analyzing the TCGA database^32^.

### Cells and reagents

Human RCC cell lines ACHN, Caki-1, Caki-2, and 786-0 cell lines were from American Type Culture Collection (ATCC) and obtained from Dr. Jonathan Pollack lab (Stanford University, Palo Alto, California). ACHN cells were cultured in DMEM; Caki-1 and Caki-2 in McCoy’s 5a Medium Modified; and 786-O in RPMI1640, all supplemented with 10% (vol/vol) fetal bovine serum (Gibco; Thermo Fischer Scientific, Inc., Grand Island, NY, USA) and with antibiotics (100 U/ml penicillin and 100 mg/ml streptomycin) in a humidified 5% CO_2_ incubator at 37 °C. Antibodies for syntaxin 6 and tubulin were from Abcam (Cambridge, MA). Enhanced chemiluminnescence (ECL) detection system was from Bio-Rad (Hercules, CA). All other reagents were obtained in their commercially available highest purity grade.

### Syntaxin-6 knockdown

ACHN cells were transfected with ON-TARGET plus human STX6 siRNA using DharmaFECT (Dharmacon; Thermo Scientific Dharmacon) as per vendor’s instruction. Briefly, siRNA was premixed and incubated with the DharmaFECT reagents and then added drop-wise on the cells. Cells were incubated for 48 hrs and syntaxin 6 mRNA was determined by real-time PCR.

### Western blotting

Cells were lysed on ice in RIPA buffer (25 mM Tris-HCL pH 7.6, 150 mM NaCI, 1% NP-40, 1% sodium deoxycholate, 0.1% SDS, 1 mM benzamidine, 2 ug·mL^−1^ leupeptin, 2 ug·mL^−1^ pepstatin A, 2 ug·mL^−1^ aprotinin and 0.5 mM PMSF) for 30 min. Protein content was measured by BCA assay (Pierce, Rockford, IL, USA). Next, 30–60 μg of protein was denatured in 4 × sodium dodecyl sulfate (SDS) loading buffer (1 mM Tris·HCl, pH 6.8, 1% SDS, 10% glycerol, 1% [vol:vol] 2-mercaptoethanol), boiled for 5 min, and subjected to SDS–polyacrylamide gel electrophoresis (PAGE) on 12% Tris–glycine gel. After blocking with 5% nonfat milk and 0.05% Tween 20 in TBS for 1 h, the membrane was first blotted for mouse monoclonal anti-syntaxin 6 antibody at a dilution of 1:200 overnight at 4 °C and then the appropriate peroxidase-conjugated secondary antibody and visualized by ECL detection system.

### Real-time PCR

The relative abundance of syntaxin 6 mRNA transcripts in cells was measured by quantitative real-time RT-PCR using TaqMan PCR. 1 µg of total RNA was reverse-transcribed in a 20 µL reaction mixture using an ABI High Capacity cDNA Archive Kit according to the manufacturer’s instruction (Applied Biosystems, Foster City, CA). The reaction contained 1x RT buffer, 100 µM of each deoxynucleoside triphosphate, 1x random primer, and 100 U of reverse transcriptase. The reaction was carried out at 25 °C for 10 min at 37 °C for 2 h. Control assays were those in which the RT enzyme or the target RNA was omitted from the reaction. Taqman PCR was performed on the cDNA samples using an ABI PRISM 7500 Sequence Detection System (Applied Biosystems). PCR was carried out in 20 µL reaction total. The thermal cycling conditions were as follows: 50 °C for 2 min, 95 °C for 10 min, 40 cycles at 95 °C for 15 sec, and 60 °C for 1 min. The relative level of expression of gene in the samples was determined using the relative 2^ΔΔCt^ expression method as previously described^33^. It was normalized against an endogenous GAPDH control. The value of the relative level of expression for the gene of interest represents two independent reactions performed in triplicate.

### MTT assay

ACHN cells (2 × 10^3^ cells/well) were plated in 96 well plates and transfected with syntaxin 6 siRNA as described above. 72 hrs later, MTT solution (Sigma) 20 ul of 5 mg/ml was added to each well and incubated for a further 4 h at 37 °C. Then the medium was removed and DMSO (200 ul) added before a further incubation of 20 min at room temperature. Finally the absorbance was measured at 560 and at 650 nm to subtract the background.

### Statistics

Statistical analysis was performed using GraphPad Prism software version 4.0 and IBM SPSS Statistics®, Version 24. Kaplan-Meier survival curves were created for overall survival. Differences between curves were compared using log-rank tests. A two-tailed calculated p value of <0.05 was considered significant. Standard Student *t* tests were used to compare continuous variables between groups and Fisher exact tests for categorical variables. We evaluated the impact of syntaxin 6 expression and other patient clinicopathologic characteristics on survival using multivariate analysis to generate Cox proportional hazard ratios for survival.

## Data Availability

The datasets generated during and/or analyzed during the current study are available from the corresponding author on reasonable request.
